# Statin Use and the Point Prevalence of Antibiotics in Ambulatory Patients with Diabetes in the National Health and Nutrition Examination Survey (NHANES) 2003–2012

**DOI:** 10.3390/antibiotics8020064

**Published:** 2019-05-27

**Authors:** Sumaiah J. Alarfaj, Alexandra Perez, Nathan R. Unger

**Affiliations:** 1 Department of Pharmaceutical Practice, Princess Nourah bint Abdulrahman University College of Pharmacy, Riyadh 11671, Saudi Arabia; 2 Department of Sociobehavioral and Administrative Pharmacy, Nova Southeastern University College of Pharmacy, Davie, FL 33328, USA; alperez@nova.edu; 3 Gilead Sciences, Foster City, CA 94404, USA; nathan.unger@gilead.com

**Keywords:** statins, point prevalence, oral antibiotics

## Abstract

In patients with diabetes, the risk of infections is increased, hypothesized to be due to alterations in the immune system, among other changes. The pleotropic effects of statins have been investigated to assess their role in reducing the risk of infection and infection-related outcomes with varying results. The aim of this study is to determine if the use of statins is associated with a decrease in the point prevalence of oral antibiotic use in ambulatory patients with diabetes. Using data from the National Health and Nutrition Examination Survey (NHANES) from 2003 to 2012, all adult patients diagnosed with diabetes were analyzed. Patients were grouped into those who were prescribed statins and those who were not. Oral antibiotic use between the two groups was compared. Data were standardized to national estimates. A total of 3240 patients with diabetes were identified, with 1575 statin users and 1665 non-statin users. After controlling for baseline socio-demographic and clinical variables, the overall point prevalence of oral antibiotic use in diabetes population was 3.5% with no difference between statin users and non-statin users (2.9% vs. 4%, *p* = 0.116). Based on the results of this study, the use of statins in patients with diabetes was not associated with a reduction in the point prevalence of antibiotic use.

## 1. Introduction 

Patients living with diabetes are at a higher risk of infections [1,2], hypothesized to be due to impaired cellular immunity, increased microorganism adherence to cells, and increased virulence of pathogens in a hyperglycemic setting [3]. The presence of diabetes doubles the risk of hospitalization from an infection and nearly doubles the infection-related mortality rate [4]. 

3-Hydroxymethylglutaryl coenzyme A (HMG-CoA) reductase inhibitors, otherwise known as statins, are indicated in select patients with diabetes for the prevention of primary and secondary cardiovascular disease in those with or without coronary heart disease [5,6]. In addition to lowering lipids, statins also exert a wide spectrum of pleiotropic effects. These effects include restoring endothelial function [7,8], increasing nitric oxide synthase expression and antioxidant activity, as well as having a host of additional anti-inflammatory and immune-modulatory properties that can potentially be compromised in patients with diabetes [9,10,11,12]. Moreover, in vitro studies reveal statins may also possess the ability to suppress bacterial growth, reduce virulence, and inhibit biofilm formation [13]. These pleiotropic effects have been examined for their benefit in patients with infection; however, investigations in hospitalized patients with sepsis, bacteremia, pneumonia, and *Clostridium difficile* infection have produced mixed results [14,15,16,17,18,19,20,21,22,23,24]. Moreover, studies investigating the effect of statin use on infection rates in patients with diabetes are limited.

With the increased use of antibiotics, estimated to be 296 million antibiotic prescriptions dispensed from outpatient pharmacies in the U.S. in 2015 [25], interventions with the potential to decrease the use of antibiotics in the community are valued. Using a population-based sample, we sought to investigate if statin use is associated with a reduction in the point prevalence of oral antibiotic use in ambulatory adults with diabetes. 

## 2. Materials and Methods 

The National Health and Nutrition Examination Survey (NHANES) uses a combination of interviews, physical exams, and laboratory tests in an effort to monitor the health status of U.S. non-institutionalized civilians. In addition to clinical, physical examination, and laboratory data, a trained interviewer collects a list of prescription medications used within the last 30 days [26]. The National Center for Health Statistics Research Ethics Review Board (ERB) approved all protocols performed by NHANES, and informed consents were obtained from all participants.

For NHANES cohorts 2003–2004 through 2011–2012, all adult patients 20 years of age or older diagnosed with diabetes at the time of the survey were included in the analysis. Using the prescription drug file, patients were grouped into two categories: those who were prescribed statins (statin users) and those who were not prescribed statins (non-statin users). Patients were included in the statin user group if they were prescribed a statin as a single ingredient (i.e., atorvastatin, cerivastatin, fluvastatin, lovastatin, pitavastatin, pravastatin, rosuvastatin, and simvastatin) or co-formulated with another medication (i.e., atorvastatin/amlodipine, lovastatin/niacin, simvastatin/ezetimibe, and simvastatin/niacin).

Antibiotics included orally administered penicillins, cephalosporins, macrolides, lincosamides, fluoroquinolones, sulfonamides, tetracyclines, and other miscellaneous antibiotics (see Appendix A). Antibiotics administered topically were excluded. NHANES does not provide an indication for the use of any medications in the prescription drug file.

The primary outcome is the point prevalence of oral antibiotic use in statin users and non-statin users. The null hypothesis is that the use of antibiotics is equivalent in statin users and non-statin users. Univariate logistic regression analyses were performed to evaluate the association between statin use and antibiotic use in ambulatory patients with diabetes (alpha = 0.05). Multivariable logistic regression analysis evaluated the effect of all the covariables including age, gender, race, Hispanic ethnicity, college education, body mass index (BMI), smoking status, number of years living with diabetes, hemoglobin A1c level, health insurance and prescription drug coverage, and number of times receiving health care services on the association of interest over the past year. Moreover, we did a subgroup analysis of antibiotic use between the two groups in patients 40 years or older and evaluated the primary outcome in subgroups according to the type of statin used and the class of antibiotic used. 

All statistical tests were performed using IBM SPSS version 23 (IBM Corp., Armonk, NY, USA). Through the use of complex sampling design, all estimates in this study are nationally representative of the civilian, non-institutionalized U.S. population of adults with diabetes and aged 20 years or older at the time of survey. 

## 3. Results

Statin use was observed in 1575 patients (48.6%) of the 3240 adult patients with diabetes identified across the five NHANES cohorts. Patient demographics are provided in Table 1. Statin users were significantly more likely to be male, white, non-smoker, obese, and older in age. Moreover, a greater proportion of statin users had health insurance, received regular health care services, and had had diabetes for more than 10 years. There was no statistically significant difference in the A1c levels between the two groups. 

**Figure 1:**
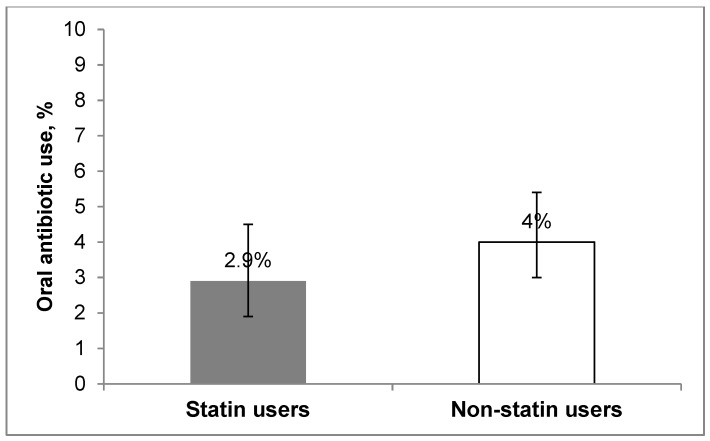
Oral antibiotic use in patients with diabetes according to statin use (*p* = 0.259 for unadjusted model, *p* = 0.116 for adjusted model).

The overall point prevalence of oral antibiotic use was 3.5% in ambulatory patients with diabetes. When evaluating the association between statin use and the point prevalence of antibiotic use in patients with diabetes, no statistically significant difference was observed in the unadjusted and adjusted models between statin users and non-statin users (2.9% (95% CI 1.8, 4.6%) vs. 4% (95% CI 3.0, 5.3%) (Figure 1). In patients 40 years of age or older, antibiotic use remained non-significant (4% vs. 3%, *p* = 0.578). Moreover, no difference in antibiotic use by antibiotic class was observed (*p* > 0.05 for all classes) as well as no difference in antibiotic use by specific statin drugs (*p* > 0.05 for all individual statins). 

## 4. Discussion

This national cohort study suggests that the use of statin in patients with diabetes is not associated with a reduction in the prevalence of oral antibiotic use. While we are not aware of any other studies that have investigated the association between statin use and antibiotic use specifically in patients with diabetes, a few cohort studies have investigated their effect on preventing infection and infection-related outcomes in this population with contrasting results. A case–control study, performed using the United Kingdom General Practice Research Database spanning 14 years, found that statin use in patients with diabetes was associated with a reduction in the risk of developing pneumonia by 50%, with community-acquired pneumonia seen in 1.1% of statin users, compared to 2.1% of non-users [27]. In Taiwan, a population-based cohort study examined the effect of statin use on lower extremity amputation after diabetic foot infection and found 52% of the 38,793 patients with diabetes were prescribed a statin. Compared to patients not prescribed a statin, a significant 52% risk reduction in lower-extremity amputations was observed (*p* < 0.01) [28]. 

Studies investigating the effect of statin use on preventing infection in the general population have yielded results similar to this study. Using outpatient and inpatient International Classification of Disease (ICD) codes, Magulick et al. compared the incidence of infections in statin users to that in non-users [29]. Of the 45,247 patients included, 29% were statin users. After adjusting for baseline variables, statin use did not decrease the incidence of bacterial (odds ratio (OR): 1.13, 95% CI: 1.06–1.19), influenza (OR: 1.06, 95% CI: 0.80–1.39), or fungal infections (OR: 0.97; 95% CI: 0.91–1.04). This is in agreement with a large meta-analysis conducted by van den Hoek et al., which included eleven randomized, placebo-controlled clinical trials totaling 30,947 subjects, 46% of whom received statin therapy [30]. Of note, the studies included in the meta-analysis were designed to evaluate efficacy of statins to prevent cardiovascular or cerebrovascular events, not infection. The overall relative risk reduction in infection-related adverse events was 1.00 (95% CI: 0.96–1.05, *p* = 0.93) compared to placebo. 

A strength of this study is the use of trained personal interviewers to gather self-reported use of the statins and antibiotics rather than using pharmacy dispensing data. This relates better with health care utilization rather than health care access. In addition, it provides a point estimate of oral antibiotic use in the U.S. population living with diabetes. The use of point prevalence in hospital antibiotic utilization has been employed previously [31,32]. While point prevalence estimations are more challenging to capture in an ambulatory population, the NHANES database provides a unique setup that may capture it with reduced error margin. 

Unfortunately, the intensity, duration, and adherence of statin treatment were not available within the NHANES database. This is important because the selection of specific statin, dose, and duration of therapy may influence outcomes. In vitro studies investigating statins as potential antimicrobials have revealed that statins differ in their spectrum of activity and impact on individual minimum inhibitory concentration (MIC) values. For example, simvastatin, atorvastatin, and rosuvastatin have demonstrated the variable ability to inhibit growth of gram-positive and gram-negative nosocomial species with antibiotic resistance, including methicillin-resistant *Staphylococcus aureus* (MRSA), vancomycin-resistant enterococci (VRE), *Pseudomonas aeruginosa*, *Acinetobacter baumannii*, or *Klebsiella pneumoniae* [33,34,35,36]. A second limitation is that NHANES did not provide indications for prescribed medications including antibiotics; therefore, we were not able to distinguish between prophylactic or treatment use of this drug category. Although this study assesses the association between statin use and the point prevalence of antibiotic use, assessing antibiotic use over a longer duration might produce more comprehensive results. 

Studies have postulated that the positive effect on therapeutic outcomes seen with preventative statin use might be attributed to a “healthy user effect” [37], that is, patients prescribed statins preventatively appear to be more apt to be up to date on immunizations, comply with prescribed therapy and lifestyle changes, as well as seek regular medical care, all of which could introduce bias to the results. In our study, statin users were significantly less likely to smoke and more likely to receive regular care for their diabetes; however, they were also more obese, had a longer time since diagnosis, and ultimately did not differ in A1c control. We were also unable to determine patient immunization status. However, statin users in this sample were significantly more likely to receive regular care, which is associated with higher vaccination rates [38]; thus, the inability to assess vaccination rates is unlikely to influence the results of this study. Regardless, differences in baseline demographics were controlled for in the multivariable analysis, likely mitigating any healthy user effect. A high quality longitudinal observational study or a randomized clinical trial will address the above-mentioned limitations and establish the causal effect of the relationship between statin and antibiotic use.

## 5. Conclusions

Based on the results of this study of several NHANES cohorts, statin use in patients with diabetes was not associated with a reduction in the point prevalence of antibiotic use. While results from this study do not provide a link between statins and a reduction in antibiotic use in patients with diabetes, it is unknown whether the use of statins in this population influenced clinical response, or hastened recovery. Additional studies are needed to confirm the lack of an impact of antibiotic use by statins in patients with diabetes.

## Figures and Tables

**Table 1:** Patient demographics.

Parameters	Overall (*n* = 3240)		Statin Users (*n* = 1575)		Non-Statin Users (*n* = 1665)		
	Estimate	SE	Estimate	SE	Estimate	SE	*p*-Value
Age, %							
20–40 years	9.2	0.6	4.1	0.7	14.1	1.1	0.000
41–60 years	40.7	1.2	36.5	1.8	44.8	1.6	
61 years and above	50.1	1.3	59.4	1.8	41.1	1.7	
Gender, %							
Female	52	1.2	49	1.7	54.9	1.4	0.003
Race/Ethnicity, %							
Hispanics	13.3	1.5	10.5	1.3	16	1.9	0.000
Whites	61.7	2.2	66.6	2	57.1	2.8	
Blacks	17.6	1.5	15.9	1.5	19.2	1.7	
Others	7.4	0.8	7.1	1	7.7	1	
Education Level, %							
High school and below	54.1	1.6	55.2	2.2	53.1	2	0.686
Some college	28.1	1.2	27.8	2	28.4	1.7	
College and above	17.8	1.2	17	1.5	18.5	1.6	
Smoking, %	34.2	1.3	27.30	2.20	41.4	2.5	0.000
BMI, %							
Normal	14	0.9	12.5	1.2	15.4	1.2	0.034
Overweight	25.5	1.1	23.8	1.5	27.3	1.7	
Obese	60.5	1.4	63.8	1.9	57.3	1.9	
Age at Diabetes Diagnosis							0.229
<20 years, %	6.3%	0.6%	5.5%	0.9%	7.1%	0.9%	
≥20 years, %	93.7%	0.6%	94.5%	0.9%	92.9%	0.9%	
A1c < 7% (53 mmol/mol), %	54	1.5	54	2	53.9	1.8	0.975
DM Years, %							
<5	33.2	1.1	29.4	1.4	36.9	1.8	0.003
5–10	28.4	0.8	28.5	1.5	28.4	1.3	
>10	38.3	1.2	42.1	1.8	34.6	1.7	
Health Insurance, %	89.7	1.6	94.1	2.1	87.1	2	0.028
Receive regular DM care, %	97.3	0.4	99.1	0.4	95.6	0.6	0.000
Prescription drug coverage ^1^, %	91.7	1	92.9	1.1	90.3	1.2	0.038

^1^ Data are not available for the 2003–2004 cohort; DM, diabetes mellitus; SE, standard error.

## Appendix A

**Table:**

Oral antibiotics listed in NHANES	
**Penicillins** Amoxicillin Amoxicillin-clavulanate Cloxacillin Dicloxacillin Penicillin V	**Cephalosporins** First Generation Cephalexin Cefadroxil Second Generation Cefuroxime Cefaclor Cefprozil Third Generation Cefdinir Cefditoren Cefixime Cefpodoxime Ceftibuten
**Macrolide Antibiotics** Azithromycin Erythromycin Clarithromycin Telithromycin	**Quinolone Antibiotics** Ciprofloxacin Norfloxacin Ofloxacin Levofloxacin Moxifloxacin Gemifloxacin
**Lincosamides** Clindamycin	**Sulfonamides** Trimethoprim-Sulfamethoxazole
**Tetracycline Antibiotics** Doxycycline Minocycline Tetracycline	**Other Antibiotics** Metronidazole Nitrofurantoin Vancomycin
